# Approaches to identify genetic variants that influence the risk for onset of fragile X-associated primary ovarian insufficiency (FXPOI): a preliminary study

**DOI:** 10.3389/fgene.2014.00260

**Published:** 2014-08-07

**Authors:** Emily G. Allen, Wendy E. Grus, Sarayu Narayan, Whitney Espinel, Stephanie L. Sherman

**Affiliations:** ^1^Department of Human Genetics, Emory UniversityAtlanta, GA, USA; ^2^Knome Inc.Cambridge, MA, USA

**Keywords:** primary ovarian insufficiency, ovarian failure, menopause, repeat expansion, disorder, epistasis

## Abstract

Fragile X-associated primary ovarian insufficiency (FXPOI) is due to an X-linked mutation that results from the expansion of a CGG repeat sequence located in the 5′ untranslated region of the *FMR1* gene (premutation, PM). About 20% of women who carry the PM have cessation of menses before age 40, a clinical condition known as premature ovarian failure (POF). This leads to a 20-fold increased risk over women in the general population. Thus, this single gene mutation has a major effect on reducing a woman's reproductive life span. Based on survival analysis of about 1300 women, we showed that the mean age at menopause among PM carriers is reduced compared with noncarriers, even after removing women who reported POF. This suggests that the majority of women with the PM, not just a subset, experience ovarian insufficiency earlier than noncarriers. To better understand the underlying mechanism of the PM and to identify genes that modify the variable expressivity of FXPOI, we conducted two pilot studies. The first focused on five common variants known to reduce age at menopause. We genotyped these SNPs in 72 women with a PM who experienced menopause and found a significant association with the total SNP risk burden and age at menopause. This suggests that these SNPs influence onset of FXPOI, after adjusting for the effect of the PM allele. In the second approach, we conducted whole genome sequencing on 10 PM carriers, five with onset of FXPOI prior to age 30 and five who experienced menopause after age 47 years. Although only a pilot study, we describe our preliminary approach to identify potential variants that may play a role in modifying onset of FXPOI and potentially play a role in idiopathic primary ovarian insufficiency. The overarching goal of both approaches is to identify predictor variants that may identify women predisposed to early onset FXPOI and to further identify genes involved in defining a woman's reproductive life span.

## Introduction

Fragile X-associated primary ovarian insufficiency (FXPOI) is one of the disorders caused by the expansion of a CGG repeat sequence located in the 5′ untranslated region (UTR) of the X-linked *FMR1* gene. About 20% of women who carry an allele with 55 to 200 unmethylated CGG repeats [termed premutation (PM) allele] develop hypergonadotropic hypogonadism and cease menstruating before age 40. This clinical condition, also known as premature ovarian failure (POF), occurs in about 1% of the general population. Thus, carrying the PM greatly increases the risk for POF (Sherman, 2000; De Caro et al., 2008; Sullivan et al., 2011).

The term primary ovarian insufficiency (POI) includes both POF and occult indicators of ovarian function, such as increased levels of follicle stimulating hormone (FSH) and decreased levels of anti-Müllerian hormone (AMH). As this entire spectrum, including the altered hormone profile, is seen among women with the PM (Murray et al., 2000; Hundscheid et al., 2001; Welt et al., 2004; Sullivan et al., 2005; Rohr et al., 2008; Spath et al., 2011a), the term “FXPOI” is well suited (Welt, 2008).

Very little is known about the mechanisms leading to FXPOI. The other clinically significant form of the *FMR1* expansion mutation is the full mutation (FM). This mutation leads to fragile X syndrome, an inherited form of intellectual and developmental disabilities. The FM is defined as an allele with >200 methylated CGG repeats and causes the silencing of the *FMR1* gene. Consequently, no or little protein product (FMRP) is made in hemizygous male carriers and reduced amounts in heterozygous female carriers. Early on, it was shown that women who carry the FM are not at increased risk for ovarian dysfunction (Schwartz et al., 1994; Allingham-Hawkins et al., 1999). This important observation indicates that reduction of FMRP does not underlie the etiology of ovarian dysfunction. Instead, some characteristic of the PM allele is the culprit. There are important molecular consequences of the PM: with increasing repeat length, there is increasing *FMR1* transcript levels and decreasing FMRP levels (Kenneson et al., 2001; Allen et al., 2004; Garcia-Alegria et al., 2007; Tassone et al., 2007; Peprah et al., 2010). Many have postulated that *FMR1* mRNA toxicity may underlie FXPOI, as is the case for the other PM-associated disorder, fragile X-associated tremor/ataxia syndrome (FXTAS) (Hagerman et al., 2001).

Not all women with the PM experience POF, only about 20%. Four factors have been examined to try to explain the incomplete penetrance of POF among PM carriers: CGG repeat length, skewed X-chromosome inactivation (XCI), smoking and background genes. First, there is a strong non-linear association of the penetrance of POF with repeat number: women with mid-range PM repeats (approximately 80–100 repeats) have the highest risk for POF. Carriers of both smaller and larger PM repeat alleles also have an increased risk of POF compared to the general population, but not to the same extent as mid-range PM repeat carriers (Sullivan et al., 2005; Ennis et al., 2006; Allen et al., 2007; Tejada et al., 2008; Spath et al., 2011b). Second, skewed XCI was considered as potentially modifying the risk of severity of FXPOI, as *FMR1* is located on the X chromosome. However, no study has found evidence for skewed XCI based on samples from fresh blood among PM carriers (Murray et al., 2000; Sullivan et al., 2005; Bione et al., 2006; Tejada et al., 2008; Rodriguez-Revenga et al., 2009; Spath et al., 2010). Assuming that XCI in blood can be used as a proxy for the correct target tissue, one possible explanation for this observation is that the toxic effect of the PM acts during a stage in development when both X chromosomes are active. Third, smoking, an important modifiable risk factor known to reduce age at menopause, has been shown to have the same effect on women with the PM as it does on noncarriers (Allen et al., 2007; Spath et al., 2011b).

Lastly, we have indirect evidence that the risk of POF depends not only on the PM allele, but also on other background genes. In Hunter et al. (2008), we used a random-effects Cox proportional hazards model to analyze age at menopause on 680 women drawn from 225 families who had a history of fragile X syndrome and 321 women from 219 families drawn from the general population. We found the presence of a statistically significant residual additive genetic effect after adjustment for *FMR1* repeat length and other covariates (*p*-values ranging between 0.0002 and 0.0027, depending on the parameterization of *FMR1* repeat size and definition of age at menopause). Using a combined data set from our U.S. study and one from the Netherlands consisting of 1068 women, we found that the mean age at menopause among first degree relatives as a predictor of age at menopause was statistically significant only among noncarriers, not among PM carriers once adjusting for PM repeat length and smoking (Spath et al., 2011b).

This indirect evidence for modifying/background variants is fortified by recent GWAS studies that have identified genetic variants associate with age at menopause (He et al., 2009; Stolk et al., 2009, 2012; Shen et al., 2013). Perry et al. (2013) also showed that these same variants were associated with age at menopause among women with early menopause (age 41–45). These results support the hypothesis that early menopause and POF may represent the tail of the menopause distribution and thus have overlapping etiology.

In addition, studies showing the molecular and biological consequences of the PM provide a basis for choosing candidate genes and pathways on which focus genetic studies. For example, proteins known to bind to the CGG track found in *FMR1* mRNA and their interacting partners are excellent candidate genes in which to study genetic variants [e.g., Pur α, hnRNP A2/B1, Sam68, miRNA processing complex DROSHA-DGCR8 (Jin et al., 2007; Sofola et al., 2007; Sellier et al., 2010, 2013)]. Another category includes genes in pathways that have been found to be altered in the FXPOI mouse model: the leutenizing hormone (LH)-mediated pathway and the mTOR signaling network (Lu et al., 2012). In this FXPOI model, LH-induced ovulation related gene expression was specifically altered. As well, there was reduced phosphorylation of Akt and mTOR proteins.

In summary, little is known about the etiology of FXPOI. Here we took three approaches to begin to determine the influence of modifying genetic variants on the onset of FXPOI. First, we examined the distribution of age at menopause among PM carriers to determine whether only a subset of PM carriers are affected with FXPOI (incomplete penetrance) or whether the age at menopause distribution is decreased among all PM carriers (variable expressivity). Next, we examined the association of five common SNPs known to reduce age at menopause in the general population among PM carriers who had experienced menopause. Lastly, we conducted an exploratory candidate gene study using whole genome sequencing (WGS) among five PM carriers with early onset FXPOI and five PM carriers with age at menopause at the typical time as controls to identify possible variants that influence onset of FXPOI.

## Materials and methods

### Study population

The study population analyzed for the three questions addressed extends that reported in Sullivan et al. (2005) and Allen et al. (2007). All participants were recruited using the same protocol. Briefly, study participants were ascertained from families with a history of FXS to enrich the sample with varying repeat lengths as well as from the general population (see Sullivan et al., 2005 for a review of ascertainment protocols). Once a proband was identified, female relatives were also invited to participate in the study. Participants were aged 18–92 and had English as their primary language. All participants were asked to complete a reproductive history questionnaire and provide a biological sample, either buccal or blood, for repeat-length determination. Subsets of this study population were used for each question addressed and are defined in more detail in Results. The protocols and consent forms for ascertainment were approved by the Institutional Review Board at Emory University.

### Data collection and variable definitions

We administered the reproductive history questionnaire to obtain demographic information including age at interview, date of birth, ethnic/racial group and education. Information on potential confounders and effect modifiers was collected and included smoking (1, ever smoked on a regular basis; 0, otherwise) and hormone use (1, current hormone use; 0, otherwise). We obtained menstrual cycle history including age at menarche and age and date of last menstrual period. If the date of last menstrual period was more than 2 months prior to the interview, we identified the cause of menses cessation. Women completed a brief medical history concerning disorders associated with ovarian dysfunction and co-morbid disorders related to reproductive aging. Although all questions were started with “Has a doctor ever told you that you had…?” all conditions were based on self-report. Medical records were not obtained to verify fertility problems or medical disorders. We defined age at menopause as the cessation of menses for at least 1 year.

### *FMR1* repeat length determination

All participants were asked to provide a biological sample, either buccal or blood. DNA was extracted using the Qiagen QiAmp DNA Blood Mini Kit (Valencia, CA). A fluorescent-sequencer method is used to determine FMR1 CGG repeat length (Meadows et al., 1996). Briefly, fluorescent-labeled primers are used to polymerase chain reaction amplify across the repeat region and the resulting product is run on an automated sequencer. Repeat lengths up to 90 can be determined with this method. In the event that a single band was detected in females, indicating either homozygous status or the presence of a larger repeat band from the second allele, an alternative polymerase chain reaction-based, hybridization technique was used (Brown et al., 1993). For heterozygous females, the CGG repeat length from the largest repeat allele was used to define *FMR1* repeat length.

### Common SNP genotyping

All individuals were genotyped for rs16991615, rs2517388, rs2277339, rs11668344, and rs365132 using predesigned TaqMan assays purchased from Applied Biosystems (Foster City, CA). Briefly, reactions were set up in a total reaction volume of 5 ul including 1× TaqMan Genotyping Master Mix, 1× TaqMan SNP Genotyping Assay probe, and 10 ng of DNA. PCR amplification was performed using Applied Biosystems' TaqMan 7900HT sequence detection software (SDS). PCR was performed by an initial activation of AmpliTaq gold (95°C for 10 min) followed by 40 cycles of denaturing (95°C for 15 s) and annealing/extension (60°C for 1 min). Alleles were automatically determined by the SDS software.

### Whole genome sequencing (WGS) and annotation

PCR-free paired end libraries were manually generated from 500 ng to 1 ug of gDNA using a modified version of TruSeq DNA v2 Sample Preparation kits (Catalog # FC-121-2001). DNA fragmentation was performed with a Covaris LE220. After fragmentation and end repair, libraries were manually size-selected to approximately 300 bp using bead-based size selection (Agencourt AMPure XP #A63881). Following size selection, libraries were A-tailed, and ligated with sequencing adapters before proceeding to library QC. Final libraries were assessed using a Caliper GX and quantified by qPCR on the Roche LightCycler 480.

Cluster generation was performed with the Illumina cBot and the TruSeq Paired End Cluster Kit (v3). Flowcells (v3) were sequenced on the Illumina HiSeq2000 platform with a 100 bp paired-end read protocol and TruSeq (v3) SBS chemistry. Read data were aligned and variants called using the CASAVA1.8.2 pipeline.

Each genome was annotated using version 2.6 of Knome's proprietary annotation engine kGAP (Knome Genome Analysis Pipeline, Knome Inc., Cambridge, MA). kGAP annotates all classes of substitution and small indel variant calls (as well as reference-matching sites and no calls) and assesses whether a call is novel or known in comparison to dbSNP135 (http://www.ncbi.nlm.nih.gov/projects/SNP/). Allele frequency information from HapMap3 (Altshuler et al., 2010) is also incorporated (additional allele frequency data are applied post-kGAP, see below). For gene context, kGAP considers variants with respect to ENSEMBL (Release 66) gene models. kGAP uses the combined SIFT/PolyPhen2 module of Condel to predict the deleteriousness of a reference-mismatching variant (Gonzalez-Perez and Lopez-Bigas, 2011). kGAP also reports conservation scores based on PhastCons scores for eutherian mammals (Siepel et al., 2005). Phenotype annotations and known literature associations applied by kGAP originate from HGMD (www.hgmd.org) or from Knome's own custom manual curation. For variant sites that overlap multiple genes, annotations are reported for all; however, prioritization is given to the gene model with the greatest predicted functional effect at the protein level.

### Identifying variants from whole genomes

Knome's proprietary kVariants software allows for the selection of the subset of kGAP-annotated variants that fit a specified mode of inheritance and/or etiology (i.e., somatic variants in tumor/normal comparisons, etc.). Within this framework, we identified confidently called variants in several candidate gene lists. Calling parameters required a site pass GATK filters in all samples (Quality = 30.0; Quality/depth = 5.0; Homoplasy run < 6; Strand bias < −0.10). Coding variants that matched the search criteria were then filtered to remove sites in known segmental duplications and to eliminate variants outside of RefSeq exons. These filtered variant lists were then further annotated to include allele frequency information from the Exome Variant Server (http://evs.gs.washington.edu/EVS/) and the 1000 Genomes Project (http://www.1000genomes.org/).

An overview of the sequencing experiment by study sample is provided in Supplement Table 1.

## Results

### Age at menopause distribution: evidence for variable expressivity

We examined the age at menopause distributions of PM carriers, including and excluding those who reported POF. This was done to test the hypothesis that there is a subset of women who are at risk for ovarian dysfunction (i.e., incomplete penetrance). The alternative hypothesis is that all women who carry the PM are at risk for some degree of ovarian dysfunction, manifesting as an overall earlier age at menopause (variable expressivity). Although these hypotheses are not necessarily mutually exclusive, they may provide insight into the types of modifying genes that might be important in understanding FXPOI.

We defined age at menopause as the cessation of menses for at least 1 year. However, pinpointing the exact age at which menopause occurs can be problematic due to the duration of the transition as well as the common use of hormone medication at the start of menopause symptoms. Here we used self-reported age at menopause without taking into account hormone use history (Scheme I in Sullivan et al., 2005). We think this may be conservative, as hormone use during transition may mask menopause and thus lead a woman to report a later age at menopause.

Among those who reported experiencing menopause, the unadjusted mean age at menopause was lowest among women who carried the mid-range PM length, as reported previously (Sullivan et al., 2005; Allen et al., 2007). When women who reported POF were excluded, the mean age at menopause increased, as expected (Table 1). To test whether the differences in mean menopause age between PM carrier groups and noncarriers were statistically significant, we estimated hazard ratios using a Cox proportional hazard model. Age at censoring for women who had not reported experiencing menopause was defined as age at interview. For those who had other reasons for cessation of menses (e.g., hysterectomy, eating disorder, etc.) were censored at age of last menses. We included the following covariates in the model: age at interview, racial/ethnic group, body mass index (BMI) and ever smoked. For each PM repeat group, the hazard ratio was significantly different from one, indicating a higher risk for reaching menopause earlier among those with the PM compare with noncarriers (Table 2).

**Table 1:** **Unadjusted mean age at menopause including and excluding women with POF**.

**Repeat group**	**Number of women**			**Including women with POF**		**Excluding women with POF**	
	**Total**	**Reported menopause/POF**	**Reported POF**	**Mean age at interview ± *SD***	**Unadjusted mean age at menopause ± *SD***	**Mean age at interview ± *SD***	**Unadjusted mean age at menopause ± *SD***
Normal: <55 repeats	694	111	6	43.4 ± 16.0	49.4 ± 4.4	43.3 ± 16.0	49.9 ± 3.8
Low PM: 55–79 repeats	247	70	11	48.1 ± 15.4	45.2 ± 5.8	47.8 ± 15.2	47.1 ± 3.0
Mid PM: 80–100 repeats	350	79	37	43.3 ± 12.8	40.5 ± 8.5	42.1 ± 12.4	46.8 ± 3.6
High PM: 100–200 repeats	113	19	5	39.5 ± 11.5	45.4 ± 6.0	38.9 ± 11.4	48.1 ± 4.4

**Table 2:** **Hazard ratios estimated from Cox proportion hazard model, adjusting for age at interview, race/ethnicity, ever smoked and BMI as covariates**.

**Repeat group**	**Including women with POF**		**Excluding women with POF**	
	**Hazard ratio (PM repeat vs. normal repeat group)**	***p*-value**	**Hazard ratio (PM repeat vs. normal repeat group)**	***p*-value**
Low PM: 55–79 repeats	2.4	<0.0001	2.3	<0.0001
Mid PM: 80–100 repeats	4.1	<0.0001	3.1	<0.0001
High PM: 100–200 repeats	2.5	0.0001	2.2	0.0035

### Common variants: influence on age at menopause among PM carriers

To examine the influence of common variants that are known to be associated with age at menopause, we chose five SNPs with the largest effect size found among women who experience normal age a menopause (Stolk et al., 2012) (Table 3). These five SNPs were also found to influence age at menopause among those with early menopause (Perry et al., 2013). All women selected for this study sample self-reported as Caucasian and had experienced menopause/FXPOI. Because this was a preliminary study with a small sample size (*n* = 72, Table 4), we conducted only one primary statistical test that combined the effect of all five SNPs. To do these we created a quantitative variable (Total SNP Risk) that equaled the sum over the five SNPs of the number of risk alleles per SNP multiplied by the effect size per allele, the effect size being that estimated from the meta-analyses of women experiencing typical age at menopause (Stolk et al., 2012; Perry et al., 2013). We then conducted linear regression with age at menopause as the outcome variable and repeat size and Total SNP Risk as predictor variables. Repeat size was parameterized as a continuous variable with the square of repeat size added in the model to account for the non-linear effect of the PM size allele. We found that Total SNP Risk, or the weighted sum of the SNP genotypes, was significantly associated with age at menopause after adjusting for effect of PM repeat length (Table 5). In this model, it explained about 7% of the variance in age at menopause (*p* = 0.03).

**Table 3:** **SNPs known to influence age at menopause (data taken from Stolk et al., 2012 and Perry et al., 2013)**.

**SNP**	**Chromosome**	**Position**	**Effect allele**	**Effect allele frequency**	**Normal menopause effect size (per allele, years)**
rs16991615	20	5896227	G	0.93	−0.948
rs11668344	19	60525476	G	0.36	−0.416
rs2277339	12	55432336	G	0.10	−0.380
rs365132	5	176311180	G	0.51	−0.287
rs2517388	8	38096889	T	0.83	−0.262

**Table 4:** **Study sample used to examine the association of common SNPs reported to influence normal age at menopause**.

	***N***	***N* with POF**	**Age at menopause Mean ± *SD***
Low PM: 55–79 repeats	25	2	45.1 ± 7.2
Mid PM: 80–100 repeats	40	14	40.4 ± 8.2
High PM: 100–200 repeats	7	1	46.0 ± 6.4

**Table 5:** **Linear regression model showing the significant association of “Total SNP Risk” variable that sums the weighted genotypes over five SNPs known to influence age at menopause (see text), adjusting for variables of PM repeat length**.

**Predictor**	***B* coefficient ± *SE***	**Partial *R*^**2**^**	***p*-value**
Repeat length	−1.650 ± 0.613	0.096	0.009
Square (repeat length)	0.009 ± 0.003	0.095	0.009
Total SNP risk	−3.710 ± 1.684	0.067	0.031

### Candidate gene study using whole genome sequencing: possible hints for further study

As a preliminary approach to identify potentially deleterious variants on a genetically susceptible background (i.e., PM allele), we conducted WGS on five women who carried the PM and had early onset FXPOI and five PM carriers who experience menopause after age 47, frequency matched by repeat size (Table 6). All women self-reported as Caucasian.

**Table 6:** **Study sample for WGS variant discovery**.

**Study ID**	**Age at menopause**	**Repeat length**	**Mean depth coverage**
**CASES**			
poi1	21	89	40.33
poi2	19	91	37.24
poi3	23	90	39.73
poi4	28	91	43.16
poi5	16	76	36.24
**CONTROLS**			
ctr6	49	90	34.78
ctr7	50	93	38.48
ctr8	50	77	35.59
ctr9	52	90	38.66
ctr10	48	90	34.12

We need to preface our results by emphasizing that this pilot study is obviously limited by the sample size. WGS identified on average 3 million variants per sample (Supplement Table 1). In a much larger study, we could interrogate each variant individually (or by gene or pathway) and ask whether a variant is associated statistically. However, even in a large study, success of a WGS study does not come from statistical significance. Instead, the main purpose of the statistical evaluation is to create a ranking of variants (or genes or pathways) for further functional studies. Nevertheless, we present preliminary results, primarily in candidate genes/pathways, to show the potential of these studies and perhaps to spark the attention of other investigators who study POI.

First we examined several categories of candidate genes: (1) genes involved in age at menopause or ovarian function based on a review of the literature (referred to as “genes of interest,” *n* = 161); (2) miRNAs found to be differentially expressed among women with POI (*n* = 13) (Baley and Li, 2012); and, based on the FXPOI mouse model, (3) genes in the mTOR signaling network as defined by KEGG (*n* = 64); and (4) genes responsive to the LH-mediated pathway (*n* = 6) (Lu et al., 2012) (Supplement Table 2). Among genes involved in age at menopause or ovarian function, we first examined common (MAF > 1%) non-synonymous variants that occurred among cases and not controls. We found 28 such variants among the 161 ovarian function genes of interest (Supplement Table 3). The P29S missense variant in *MSH5* (8% in general population) has been observed in women with POI (Mandon-Pepin et al., 2008) and occurred in a women (poi2) whose onset of FXPOI was age 19. Of the six variants found in multiple cases, none was predicted to change function.

We also identified rare (MAF < 1%) or novel non-synonymous variants among cases and not controls in our 161 genes of interest. We found 21 variants, with three that are predicted to change function in the following genes: *IGF2R, NBN, TNFRSF17* (Supplement Table 4).

Among the miRNAs that were reported to be differentially expressed in POI cases (*n* = 13) (Baley and Li, 2012), no case-specific variants were identified. There were four variants among those 13 miRNAs and all appeared among both cases and controls (Supplement Table 5).

Findings from the FXPOI mouse model suggested that the PM allele may disrupt mTOR/AKT signaling and may alter LH-induced ovulation related gene expression (Lu et al., 2012). Thus, we examined variation in the 64 genes in the KEGG mTOR signaling pathway (hsa04150) to play a role in this signaling network and in the LH-mediated pathway (Supplement Table 6). We identified common case-specific non-synonymous variants in three genes: *PIK3CD, TSC2*, and *EREG*. Interestingly, *EREG* was one of the mRNAs shown to be down-regulated in FXPOI mice (Lu et al., 2012). We also found four rare case-specific non-synonymous variants in the following genes: *PIK3CB, PIK3CG, CAB39l, TSC2* [Note that the novel variant in *TSC2* is the same variant that is listed among the genes of interest (Supplement Table 2)].

Lastly, we took a more agnostic approach and we asked whether there were any genes enriched for variants among cases compared with controls. To do this, we identified non-synonymous variants that were specific to cases or to controls. We then selected genes with at least five case-specific variants and where there were at least twice as many case-specific variants as control-specific variants. We then filtered out genes that are known to harbor frequent variants due to extreme length, relaxed selection, or likely false positives due to the presence of a cryptic paralog. In all, there were 25 genes enriched for variants in cases (Supplement Table 7). Among these, two are interesting in this context: (1) *AKAP1* encodes an A-kinase anchor proteins that anchors PKA to mitochondria; correct localization of PKA is required for oocyte maturation (Newhall et al., 2006; Nishimura et al., 2013) and (2) *GREB1* encodes an early response gene in the estrogen-regulated pathway and is involved in hormone-dependent breast cancer cell growth (Rae et al., 2005).

## Discussion

FXPOI is the leading known cause of POI and can be identified through genetic testing, prior to symptoms of POI. Approximately 11% of women with familial POF and about 3% of those with isolated POF carry the PM and are diagnosed with FXPOI (Murray et al., 1998; Marozzi et al., 2000; Mallolas et al., 2001; Bussani et al., 2004). PM carrier frequency in the general population is estimated at 0.40–0.67% of women (Cronister et al., 2008; Seltzer et al., 2012) The course of FXPOI is variable with respect to severity. Currently, women identified with the PM provide one of few opportunities to examine modifying genetic variants and environmental factors in the context of a single gene mutation that increases severity of POI. The findings from the FXPOI model are highly likely to translate to other forms of POI.

The *FMR1* PM allele significantly reduces a woman's reproductive life span, especially among those who carry alleles with about 80–100 repeats. Not only does this major gene effect interfere with fertility, the most immediate and significant consequence of diminished ovarian function, but it also increases the risk for other disorders related to hypoestrogenism at an early age. These include an increased risk for low bone density, earlier onset osteoporosis and fractures (Gallagher, 2007), impaired endothelial function (Kalantaridou et al., 2004), earlier onset of coronary heart disease (Atsma et al., 2006), and increased cardiovascular mortality and overall mortality (e.g., Jacobsen et al., 2003; Mondul et al., 2005). Also, women with POI are reported to have more symptoms of psychological distress than women with normal ovarian function (e.g., van der Stege et al., 2008). Hypoestrogenism has cognitive consequences as well. Estrogen is a neuro-protective agent which plays an important role in brain functioning, and changes in estrogen levels during aging are associated with reduced cognitive function and an increased risk of Alzheimer's disease (Janicki and Schupf, 2010). For women who carry the PM and, thus, are at risk for the neurodegenerative disorder, FXTAS, early estrogen deficiency could be particularly detrimental. Thus, identifying genes that predispose to early onset FXPOI is clinically significant.

In this preliminary report, we chose two approaches to identify genes that may explain the variable expressivity of FXPOI. We first choose genetic variants known to reduce age at menopause in women who experience menopause at the typical age (Stolk et al., 2012) and in those who experience early menopause (Perry et al., 2013). We found preliminary evidence that the “burden” of these five SNPs was statistically significantly associated with age at menopause among women who carry the PM, after adjusting for repeat size. However, our sample size was limited and a replication study needs to be conducted before strong conclusions can be drawn. Nevertheless, these findings suggest that genetic variants that explain some of the heritability of age at menopause may also influence ovarian function on the background of a major gene effect of the PM.

Advances on several technical and analytical fronts have made WGS a viable approach. It allows a comprehensive way to identify the full spectrum of genetic variation. We took this approach to compare the variation among women in the extreme tails of the age of onset distribution for FXPOI. Although we were only able to study five women in each tail of the age distribution in this pilot study, it is a start to an important approach to identify rare variants that may influence the severity of FXPOI and potentially identify novel genes involved in idiopathic POI. The Supplement Tables generated from this results list many case-specific potentially damaging variants that might complement studies of other investigators. However, on there own, no conclusions can be drawn. Clearly a larger study is needed to further delineate a list of candidate variants and pathways, followed by functional studies to confirm that such variants disrupt ovarian function.

## Author contributions

Emily G. Allen was involved in the study design, data acquisition and led the data analysis. Wendy E. Grus conducted the bioinformatic analyses to prioritize genetic variants from the whole genome sequencing experiment. Sarayu Narayan was involved in the sample preparation, SNP genotyping, and performing the association studies of the SNP data set. Whitney Espinel was involved in the analysis of the age at menopause to examine variable expressivity of FXPOI. Stephanie L. Sherman was involved in the study design and implementation. All were involved in results interpretation and writing of the manuscript.

### Conflict of interest statement

The authors declare that the research was conducted in the absence of any commercial or financial relationships that could be construed as a potential conflict of interest.
