# Supplementary material for: Approaches to identify genetic variants that influence the risk for onset of fragile X-associated primary ovarian insufficiency (FXPOI): a preliminary study
Source: Front Genet. 2014 Aug 7;5:260. doi: 10.3389/fgene.2014.00260 (PMC4124461; doi:10.3389/fgene.2014.00260)
Supplement: Supplementary file 1 [file DataSheet1.DOCX]

| **Supplement Table 1. Characteristics of the whole genome sequencing experiment by sample.** | | | | | | | | | | |
| --- | --- | --- | --- | --- | --- | --- | --- | --- | --- | --- |
|  | **Poi1** | **Poi2** | **Poi3** | **Poi4** | **Poi5** | **Ctr6** | **Ctr7** | **Ctr8** | **Ctr9** | **Ctr10** |
| **Sequence coverage/quality measures** | | | | | | | | | | |
| Reference genome length | 3095693981 | 3095693981 | 3095693981 | 3095693981 | 3095693981 | 3095693981 | 3095693981 | 3095693981 | 3095693981 | 3095693981 |
| Proportion unique reads | 0.9802 | 0.9789 | 0.9839 | 0.9810 | 0.9803 | 0.9807 | 0.9818 | 0.9807 | 0.9787 | 0.9809 |
| Proportion duplicate reads | 0.0198 | 0.0211 | 0.0161 | 0.0190 | 0.0197 | 0.0193 | 0.0182 | 0.0193 | 0.0213 | 0.0191 |
| Mean depth coverage | 40.3342 | 37.2426 | 39.7254 | 43.1623 | 36.2406 | 35.5909 | 38.4821 | 34.1221 | 38.6608 | 34.7796 |
| **Sequence variation measures** | | | | | | | | | | |
| **Total sites called (out of 3,095,693,981)** | **2783644911** | **2782823839** | **2784352063** | **2784517530** | **2782208933** | **2782117322** | **2783170930** | **2782007559** | **2783003963** | **2781645005** |
| Proportion called | 0.8992 | 0.8989 | 0.8994 | 0.8995 | 0.8987 | 0.8987 | 0.8990 | 0.8987 | 0.8990 | 0.8986 |
| Called heterozygous | 3313308 | 3243308 | 3268752 | 3371370 | 3132392 | 3167893 | 3183235 | 3173104 | 3309174 | 3155451 |
| Proportion called heterozygous | 0.0012 | 0.0012 | 0.0012 | 0.0012 | 0.0011 | 0.0011 | 0.0011 | 0.0011 | 0.0012 | 0.0011 |
| Ti/Tv Ratio | 2.0586 | 2.0593 | 2.0558 | 2.0545 | 2.0646 | 2.0598 | 2.0601 | 2.0647 | 2.0575 | 2.0625 |
| **Total sites called reference-mismatching** | 5050240 | 5026950 | 5037515 | 5074520 | 4949945 | 4960824 | 4972231 | 4965990 | 5056420 | 4959710 |
| Known | 4374833 | 4351226 | 4374026 | 4403834 | 4285854 | 4306510 | 4318427 | 4305048 | 4389383 | 4304117 |
| Homozygous known | 1654683 | 1671043 | 1668301 | 1645684 | 1635509 | 1631953 | 1651175 | 1640176 | 1653946 | 1653699 |
| Ti/Tv known | 2.0791 | 2.0798 | 2.0749 | 2.0761 | 2.0846 | 2.0784 | 2.0803 | 2.0843 | 2.0782 | 2.0821 |
| Novel | 413723 | 415613 | 414585 | 418907 | 377158 | 382925 | 390663 | 385423 | 407745 | 381406 |
| Homozygous novel | 107008 | 105475 | 105779 | 106998 | 99664 | 101930 | 103545 | 100532 | 105301 | 103511 |
| Ti/Tv novel | 1.3628 | 1.3975 | 1.4021 | 1.3267 | 1.3166 | 1.3548 | 1.3223 | 1.3444 | 1.3277 | 1.3247 |
| Synonymous | 11926 | 11786 | 11843 | 11890 | 11730 | 11708 | 11604 | 11833 | 11762 | 11715 |
| Homozygous synonymous | 4431 | 4534 | 4422 | 4428 | 4434 | 4375 | 4415 | 4387 | 4389 | 4392 |
| Missense | 11768 | 11834 | 11768 | 11874 | 11571 | 11559 | 11474 | 11674 | 11808 | 11604 |
| Homozygous missense | 4304 | 4459 | 4398 | 4355 | 4258 | 4326 | 4301 | 4215 | 4330 | 4233 |
| Nonsense | 107 | 126 | 123 | 133 | 119 | 120 | 103 | 120 | 116 | 136 |
| Homozygous nonsense | 28 | 30 | 31 | 25 | 23 | 23 | 19 | 23 | 30 | 28 |
| Read-through | 50 | 46 | 45 | 46 | 55 | 50 | 51 | 47 | 57 | 50 |
| Homozygous read-through | 19 | 22 | 24 | 21 | 19 | 19 | 24 | 19 | 23 | 18 |
| Indel | 1056631 | 1038536 | 1037456 | 1077041 | 988117 | 1005860 | 1017796 | 998380 | 1057822 | 1004967 |
| Triplet (frame-preserving) indel | 1914 | 1901 | 1824 | 1979 | 1956 | 1907 | 1859 | 1804 | 1986 | 1846 |
| Homozygous triplet indel | 647 | 637 | 589 | 632 | 678 | 655 | 640 | 661 | 677 | 685 |
| Frameshift indel | 516 | 480 | 498 | 527 | 527 | 512 | 489 | 463 | 528 | 504 |
| Homozygous frameshift indel | 239 | 244 | 244 | 255 | 234 | 247 | 236 | 200 | 262 | 237 |
| Splice region (<=5b from splice site) | 2206 | 2175 | 2171 | 2199 | 2194 | 2199 | 2200 | 2151 | 2236 | 2188 |
