# Supplementary material for: Approaches to identify genetic variants that influence the risk for onset of fragile X-associated primary ovarian insufficiency (FXPOI): a preliminary study
Source: Front Genet. 2014 Aug 7;5:260. doi: 10.3389/fgene.2014.00260 (PMC4124461; doi:10.3389/fgene.2014.00260)
Supplement: Supplementary file 2 [file DataSheet2.DOCX]

| **Supplement Table 2. List of candidate genes on which analyses were focused: 1) genes involved in age at menopause or ovarian function (referred to as genes of interest, n=161); 2) miRNAs found to be differentially expressed among women with POI (n=13)(Baley and Li, 2012); and, based on the FXPOI model, 3) genes in the mTOR signaling network as defined by KEGG (n=64); and 4) genes responsive to the LH-mediated pathway (n=6)(Lu et al., 2012).** | | | | | | | |
| --- | --- | --- | --- | --- | --- | --- | --- |
| **Age at menopause/ovarian function genes of interest** | | | **miRNAs differentially expressed among women with POI** | **mTOR signaling network** | | **LH-mediated pathway** | |
| *ADAMTS19* | *FSHR* | *RNF44* | let-7c | *AKT1* | | *AREG* | |
| *AIRE* | *FST* | *RPA2* | miR125b2* | *AKT1S1* | | *EDN2* | |
| *ALOX12* | *GALT* | *RPL10* | miR139-3P | *AKT2* | | *EREG* | |
| *AMH* | *GDF9* | *RSPO1* | miR144 | *AKT3* | | *HAS2* | |
| *AMHR2* | *GDNF* | *RUNDC2A* | miR146 | *BRAF* | | *IL6* | |
| *ANKK1* | *GHR* | *SALL4* | miR146a | *CAB39* | | *PTGS2* | |
| *AR* | *GSPT1* | *SMAD1* | miR196a2 | *CAB39L* | |  |  |
| *ASH2L* | *HDC* | *SMAD3* | miR202 | *DDIT4* | |  |  |
| *BAX* | *HELQ* | *SMAD5* | miR23a | *EIF4B* | |  |  |
| *BCL2L1* | *HK3* | *SMAD7* | miR27a | *EIF4E* | |  |  |
| *BDNF* | *IGF1* | *SMPDL3B* | miR654-5P | *EIF4E1B* | |  |  |
| *BICD* | *IGF2R* | *SOHLH1* | miR765 | *EIF4E2* | |  |  |
| *BMP15* | *INHA* | *SOHLH2* | miRNA342-3P | *EIF4EBP1* | |  |  |
| *BMP2* | *INHBA* | *SPO11* |  | *FIGF* | |  |  |
| *BMP4* | *INHBB* | *SRD5A1* |  | *HIF1A* | |  |  |
| *BMP7* | *KIT* | *STRAP* |  | *IGF1* | |  |  |
| *BMP8B* | *KITLG* | *SYCP2L* |  | *IKBKB* | |  |  |
| *BMPR1A* | *KPNA3* | *TAF4B* |  | *INS* | |  |  |
| *BMPR1B* | *LHR* | *TDRD3* |  | *IRS1* | |  |  |
| *BMPR2* | *LHX8* | *TGFBR1* |  | *MAPK1* | |  |  |
| *BRSK1* | *LHX9* | *TIAR* |  | *MAPK3* | |  |  |
| *C11ORF46* | *LSH* | *TLK1* |  | *MLST8* | |  |  |
| *C3ORF38* | *MCM8* | *TMEM150B* |  | *MTOR* | |  |  |
| *CDKN1A* | *MGAT1* | *TMEM35* |  | *PDPK1* | |  |  |
| *CGGBP1* | *MOS* | *TNF* |  | *PGF* | |  |  |
| *CNOT6* | *MPPED2* | *TNFRSF17* |  | *PIK3CA* | |  |  |
| *CONNEXIN 37* | *MSH4* | *TSC2* |  | *PIK3CB* | |  |  |
| *CONNEXIN 43* | *MSH5* | *UIMC1* |  | *PIK3CD* | |  |  |
| *CPEB1* | *MSY2* | *WNT4* |  | *PIK3CG* | |  |  |
| *CXCL12* | *NANOS3* | *XPNPEP2* |  | | *PIK3R1* |  |  |
| *CXCR4* | *NBN* | *ZAR1* |  | *PIK3R2* | |  |  |
| *CXCR7* | *NGF* | *ZFX* |  | *PIK3R3* | |  |  |
| *CYP19A1* | *NLRP11* | *ZNF654* |  | *PIK3R5* | |  |  |
| *CYP1B1* | *NOBOX* |  |  | *PRKAA1* | |  |  |
| *DAZAP1* | *NOG* |  |  | *PRKAA2* | |  |  |
| *DAZL* | *NR5A1* |  |  | *PRKCA* | |  |  |
| *DIAPH2* | *NRF2* |  |  | *PRKCB* | |  |  |
| *DICER* | *NT4* |  |  | *PRKCG* | |  |  |
| *DMC1* | *NTRK1* |  |  | *PTEN* | |  |  |
| *DNAJC8* | *NTRK2* |  |  | *RHEB* | |  |  |
| *DNMT1* | *NXF5* |  |  | *RICTOR* | |  |  |
| *EIF2B1* | *OCT4* |  |  | *RPS6* | |  |  |
| *EIF2B2* | *P27KIP1* |  |  | *RPS6KA1* | |  |  |
| *EIF2B3* | *PDPK1* |  |  | *RPS6KA2* | |  |  |
| *EIF2B4* | *PGR* |  |  | *RPS6KA3* | |  |  |
| *EIF2B5* | *PGRMC1* |  |  | *RPS6KA6* | |  |  |
| *EIF2C2* | *PIN1* |  |  | *RPS6KB1* | |  |  |
| *EPB41L5* | *PMM2* |  |  | *RPS6KB2* | |  |  |
| *EPS8* | *POF1B* |  |  | *RPTOR* | |  |  |
| *ESR1* | *POG* |  |  | *RRAGA* | |  |  |
| *EXO1* | *POLG* |  |  | *RRAGB* | |  |  |
| *FANCA* | *PPARG* |  |  | *RRAGC* | |  |  |
| *FANCC* | *PRIM1* |  |  | *RRAGD* | |  |  |
| *FANCG* | *PRRC2A* |  |  | *STK11* | |  |  |
| *FGF8* | *PTEN* |  |  | *STRADA* | |  |  |
| *FIGLA* | *PTHB1* |  |  | *TNF* | |  |  |
| *FMR1* | *PTPRO* |  |  | *TSC1* | |  |  |
| *FMR2* | *RAD51C* |  |  | *TSC2* | |  |  |
| *FNDC4* | *RALB* |  |  | *ULK1* | |  |  |
| *FOXE1* | *RAP80* |  |  | *ULK2* | |  |  |
| *FOXL2* | *REC8* |  |  | *ULK3* | |  |  |
| *FOXO1A* | *RERG* |  |  | *VEGFA* | |  |  |
| *FOXO3A* | *RFPL4* |  |  | *VEGFB* | |  |  |
| *FSHB* | *RHBDL2* |  |  | *VEGFC* | |  |  |

**Reference from Supplement Tables:**

Baley, J., and Li, J. (2012). MicroRNAs and ovarian function. *Journal of ovarian research* 5**,** 8. doi: 10.1186/1757-2215-5-8.

Lu, C., Lin, L., Tan, H., Wu, H., Sherman, S.L., Gao, F., Jin, P., and Chen, D. (2012). Fragile X premutation RNA is sufficient to cause primary ovarian insufficiency in mice. *Human molecular genetics* 21**,** 5039-5047. doi: 10.1093/hmg/dds348.
