# Supplementary material for: Approaches to identify genetic variants that influence the risk for onset of fragile X-associated primary ovarian insufficiency (FXPOI): a preliminary study
Source: Front Genet. 2014 Aug 7;5:260. doi: 10.3389/fgene.2014.00260 (PMC4124461; doi:10.3389/fgene.2014.00260)
Supplement: Supplementary file 3 [file DataSheet3.DOCX]

| **Supplement Table 3. Common non-synonymous variants (MAF>1%) found among cases and not controls in 161 genes of interest.** (“Call confidence” is the lowest call confidence among all 10 subjects; sCondel score is based on PolyPhen2 and SIFT.) | | | | | | | | | | | |
| --- | --- | --- | --- | --- | --- | --- | --- | --- | --- | --- | --- |
| **Chr** | **Position** | **in subject(s)** | **Call confi-dence** | **Variant class** | **Condel score** | **Conser-**  **vation**  **score** | **Gene** | **Residue change** | **MAF % - Eur, EVS** | **MAF % - Eur, 1KG** | **dbSNP ID** |
| 1 | 38079516 | poi4 | 127 | missense | 0.793 | 1 | *RSPO1* | K162Q | 5.7 | 4 | rs36043533 |
| 1 | 76345822 | poi3 | 65 | missense | 0.783 | 1 | *MSH4* | Y589C | 0.9 | 2 | rs5745459 |
| 1 | 76363755 | poi2, poi3 | 69 | missense | 0.024 | 1 | *MSH4* | E840D | 1.1 | 1 | rs114093703 |
| 1 | 242023897 | poi5 | 87 | missense | 0.726 | 1 | *EXO1* | N279S | 3.4 | 4 | rs4149909 |
| 1 | 242048679 | poi3 | 72 | missense | 0.492 | 0.021 | *EXO1* | G759E | 0.9 | 2 | rs4150001 |
| 3 | 183954202 | poi2, poi4 | 127 | missense | 0.366 | 0.063 | *VWA5B2* | A210T | 3.4 | 3 | rs61744881 |
| 3 | 183963380 | poi3 | 127 | missense | 0.016 | 1 | *ALG3* | I107V | 1.8 | 1 | rs2233463 |
| 3 | 183994446 | poi5 | 127 | missense | 0.426 | 0.046 | *ECE2* | T73I | 2.8 | 4 | rs13063766 |
| 3 | 184019680 | poi1, poi2, poi4, poi5 | 119 | missense | 0.012 | 0.259 | *PSMD2* | A176T | 4.9 | 4 | rs11545172 |
| 5 | 176316558 | poi3 | 127 | missense | 0.945 | 0.97 | *HK3* | G246D | 2.2 | 2 | rs61749653 |
| 5 | 176409573 | poi4 | 101 | missense | 0.896 | 0.716 | *UIMC1* | R15W | 1.5 | 2 | rs13167812 |
| 6 | 10874904 | poi3 | 127 | missense | 0.572 | 0 | *GCM2* | Y282D | 1.2 |  |  |
| 6 | 31592063 | poi5 | 125 | missense |  | 1 | *PRRC2A* | P106L | 5.9 |  |  |
| 6 | 31601734 | poi3 | 82 | missense | 0.522 | 1 | *PRRC2A* | R1546Q | 4.2 |  |  |
| 6 | 31603044 | poi2 | 127 | missense | 0.729 | 0.996 | *PRRC2A* | D1749G | 1.9 |  |  |
| 6 | 31603188 | poi2 | 127 | missense | 0.889 | 0.977 | *PRRC2A* | V1768M | 2.7 |  |  |
| 6 | 31708327 | poi2 | 106 | missense | 0.445 | 0 | *MSH5* | P29S | 8.3 |  |  |
| 6 | 31731880 | poi1, poi2 | 120 | missense |  | 0.014 | *C6orf26* | P99L | 12.2 |  |  |
| 10 | 88635778 | poi1, poi2, poi4 | 108 | missense |  | 1 | *BMPR1A* | P2T | 25.6 | 25 | rs11528010 |
| 11 | 100999244 | poi2 | 127 | missense | 0.911 | 0.84 | *PGR* | P186L | 1.2 | 1 |  |
| 12 | 12871098 | poi2, poi4 | 112 | missense | 0.143 | 0.977 | *CDKN1B* | V109G | 23.8 | 23 | rs2066827 |
| 12 | 15656845 | poi5 | 108 | missense | 0.825 | 1 | *PTPRO* | N370K | 2.7 | 3 | rs61754411 |
| 15 | 50555543 | poi1 | 127 | missense | 0.365 | 0.893 | *HDC* | T31M | 11.3 | 10 | rs17740607 |
| 15 | 51507967 | poi1 | 118 | missense | 0.383 | 0.819 | *CYP19A1* | R264C | 3.3 | 3 | rs700519 |
| 16 | 2110794 | poi5 | 127 | missense | 0.746 | 0.441 | *TSC2* | R367Q | 2.0 | 1 | rs1800725 |
| 17 | 56798127 | poi1 | 127 | missense | 0.872 | 1 | *RAD51C* | T287A | 1.0 | 1 | rs28363317 |
| 20 | 5933107 | poi1 | 113 | missense | 0.089 | 0.43 | *MCM8* | Q63K | 11.8 | 13 | rs236110 |
| 20 | 50406629 | poi1 | 123 | missense | 0.002 | 0.196 | *SALL4* | I798L | 5.8 | 6 | rs6091375 |
