# Supplementary material for: Approaches to identify genetic variants that influence the risk for onset of fragile X-associated primary ovarian insufficiency (FXPOI): a preliminary study
Source: Front Genet. 2014 Aug 7;5:260. doi: 10.3389/fgene.2014.00260 (PMC4124461; doi:10.3389/fgene.2014.00260)
Supplement: Supplementary file 4 [file DataSheet4.DOCX]

| **Supplement Table 4. Rare (MAF<1%) or novel non-synonymous variants found among cases and not controls in 161 genes of interest.** (“Call confidence” is the lowest call confidence among all 10 subjects; “Novel” refers to a variant that is not found in dbSNP135, HAPMAP3, 1000 Genomes or EVS; Condel score is based on PolyPhen2 and SIFT.) | | | | | | | | | | | | |
| --- | --- | --- | --- | --- | --- | --- | --- | --- | --- | --- | --- | --- |
| **Chr** | **Position** | **in subject(s)** | **Call confi-dence** | **Novel** | **Variant class** | **Condel score** | **Conser-vation score** | **Gene** | **Residue change** | **MAF % - Eur, EVS** | **MAF % - Eur, 1KG** | **dbSNP ID** |
| 1 | 156830778 | poi4 | 107 |  | missense | 0.673 | 0.01 | *NTRK1* | G18E | 0.7 |  |  |
| 1 | 242016739 | poi4 | 102 | Y | missense | 0.804 | 0.048 | *EXO1* | R121Q |  |  |  |
| 2 | 120776741 | poi1 | 127 |  | missense | 0.945 | 0.051 | *EPB41L5* | R28C | 0.6 | 0.4 |  |
| 4 | 55593430 | poi1 | 117 |  | missense | 0.011 | 0.284 | *KIT* | V530I | 0.1 | 0.1 | rs72550822 |
| 6 | 10784716 | poi1 | 108 |  | missense | 0.241 | 0.001 | *MAK* | E469K | 0.01 |  |  |
| 6 | 31595772 | poi1, poi2, poi4 | 116 |  | 12-bp del |  | 1 | *PRRC2A* |  |  |  |  |
| 6 | 160482641 | poi5 | 127 |  | missense | 0.751 | 0.596 | *IGF2R* | V1124I | 0.01 |  |  |
| 6 | 160494927 | poi1 | 122 |  | missense | 0.307 | 1 | *IGF2R* | Q1696R | 0.02 |  |  |
| 8 | 90990520 | poi1 | 99 |  | missense | 0.935 | 1 | *NBN* | I89V | 0.2 | 0.1 |  |
| 9 | 35077022 | poi3 | 127 | Y | missense | 0.756 | 0.326 | *FANCG* | P241L |  |  |  |
| 9 | 138589431 | poi2 | 127 | Y | missense | 0.231 | 0 | *SOHLH1* | E129D |  |  |  |
| 11 | 113265723 | poi1 | 127 |  | missense | 0.235 | 0.948 | *ANKK1* | R185Q | 0.1 | 0.1 | rs115800217 |
| 12 | 15654573 | poi1 | 114 |  | missense | 0.403 | 1 | *PTPRO* | I228V | 0.3 | 1 | rs117540301 |
| 14 | 95562994 | poi4 | 109 | Y | 3-bp del |  | 0.96 | *DICER1* |  |  |  |  |
| 16 | 2134981 | poi2 | 122 |  | 3-bp del |  | 0.985 | *TSC2* |  |  |  |  |
| 16 | 12009530 | poi2, poi3, poi4 | 43 | Y | 3-bp ins |  | 0.006 | *GSPT1* |  |  |  |  |
| 16 | 12061594 | poi2 | 127 |  | missense | 0.817 | 1 | *TNFRSF17* | M149R | 0.1 |  |  |
| 16 | 89836977 | poi3 | 127 |  | missense | 0.935 | 0.338 | *FANCA* | P739L | 0.1 |  | rs45441106 |
| 21 | 45713725 | poi1 | 127 | Y | missense | 0.935 | 0.294 | *AIRE* | R248W |  |  |  |
| X | 66766356 | poi3 | 20 | Y | 39-bp del |  | 0.06 | *AR* |  |  |  |  |
| X | 84600969 | poi3 | 96 |  | missense | 0.004 | 0.028 | *POF1B* | P207S |  |  | rs363766 |
