# Supplementary material for: Approaches to identify genetic variants that influence the risk for onset of fragile X-associated primary ovarian insufficiency (FXPOI): a preliminary study
Source: Front Genet. 2014 Aug 7;5:260. doi: 10.3389/fgene.2014.00260 (PMC4124461; doi:10.3389/fgene.2014.00260)
Supplement: Supplementary file 5 [file DataSheet5.DOCX]

| **Supplement Table 5. Variants identified among 13 miRNAs reported to be differentially expressed among women with POI.** (“Call confidence” is the lowest call confidence among all 10 subjects) | | | | | |
| --- | --- | --- | --- | --- | --- |
| **Chr** | **Position** | **in subject(s)** | **Call confidence** | **Conser-vation score** | **Gene** |
| 5 | 159912417 | poi3, poi4, ctr7, ctr10 | 127 | 1 | *MIR146A* |
| 10 | 135061111 | poi4, ctr7, ctr10 | 54 | 0.001 | *MIR202* |
| 12 | 54385598 | poi1, poi2, poi3, poi4, poi5, ctr6, ctr7, ctr8, ctr10 | 127 | 1 | *MIR196A2* |
| 19 | 13947291 | poi3, poi4, ctr7, ctr9, ctr10 | 127 | 0.159 | *MIR27A* |
