# Supplementary material for: Approaches to identify genetic variants that influence the risk for onset of fragile X-associated primary ovarian insufficiency (FXPOI): a preliminary study
Source: Front Genet. 2014 Aug 7;5:260. doi: 10.3389/fgene.2014.00260 (PMC4124461; doi:10.3389/fgene.2014.00260)
Supplement: Supplementary file 6 [file DataSheet6.DOCX]

| **Supplement Table 6: a) Common (MAF>1%) and b) rare (MAF<1%) or novel case-specific non-synonymous variants found in genes that are part of the mTOR/AKT signaling pathway as defined by KEGG or are in the LH-mediated pathway.** (“Call confidence” is the lowest call confidence among all 10 subjects; “Novel” refers to a variant that is not found in dbSNP135, HAPMAP3, 1000 Genomes or EVS; Condel score is based on PolyPhen2 and SIFT.) | | | | | | | | | | | | | |
| --- | --- | --- | --- | --- | --- | --- | --- | --- | --- | --- | --- | --- | --- |
| **a) COMMON VARIANTS** | | | | | | | | | | | | | |
| **Chr** | **Position** | **in subject(s)** | | **Call**  **confi-dence** | **Novel** | **Variant class** | **Con-del score** | **Conser-vation**  **score** | **Gene** | **Residue change** | **MAF % - Eur, EVS** | **MAF % - Eur, 1KG** | **dbSNP ID** |
| 1 | 9777598 | poi3 | | 127 | -- | missense | 0.629 | 0.985 | *PIK3CD* | S312C | 3.0 | 3 | rs61755420 |
| 4 | 75248504 | poi1 | | 111 | -- | missense | 0.462 | 1 | *EREG* | C141F | 5.5 | 5 | rs78803121 |
| 16 | 2110794 | poi5 | | 127 | -- | missense | 0.746 | 0.441 | *TSC2* | R367Q | 2.0 | 1 | rs1800725 |
| **b) RARE VARIANTS (not observed in 1000 Genomes or dbSNP)** | | | | | | | | | | | | | |
| 3 | 138400809 | poi3 | 99 | | Y | missense | 0.945 | 1 | *PIK3CB* | R835W |  |  |  |
| 7 | 106508417 | poi5 | 127 | |  | missense | 0.699 | 1 | *PIK3CG* | V138M | 0.1 |  |  |
| 13 | 49951230 | poi3 | 66 | | Y | missense | 0.945 | 1 | *CAB39L* | K50E |  |  |  |
| 16 | 2134981 | poi2 | 122 | |  | 3-bp del |  | 0.985 | *TSC2* |  |  |  |  |
