# Supplementary material for: Approaches to identify genetic variants that influence the risk for onset of fragile X-associated primary ovarian insufficiency (FXPOI): a preliminary study
Source: Front Genet. 2014 Aug 7;5:260. doi: 10.3389/fgene.2014.00260 (PMC4124461; doi:10.3389/fgene.2014.00260)
Supplement: Supplementary file 7 [file DataSheet7.DOCX]

| **Supplement Table 7. Genes in which variants are enriched among cases compare with controls (see text for filtering methods).** | | |
| --- | --- | --- |
| **Gene** | **N of variants in cases** | **N of variants in controls** |
| *UGT1A5* | 9 | 0 |
| *DCP1B* | 7 | 0 |
| *SLC35G4* | 7 | 0 |
| *HLA-DPB1* | 6 | 0 |
| *PLEKHG5* | 6 | 0 |
| *PTX4* | 6 | 0 |
| *ZSWIM2* | 6 | 0 |
| ***AKAP1*** | **5** | **0** |
| *AZI1* | 5 | 0 |
| *BTNL3* | 5 | 0 |
| *DSG1* | 5 | 0 |
| *GPR98* | 5 | 0 |
| *PCF11* | 5 | 0 |
| *POM121L2* | 5 | 0 |
| *ZNF85* | 5 | 0 |
| *NBPF3* | 6 | 1 |
| *GPR179* | 5 | 1 |
| *EYS* | 9 | 2 |
| *ACAN* | 6 | 2 |
| *COL20A1* | 5 | 2 |
| ***GREB1*** | **5** | **2** |
| *GRHL3* | 5 | 2 |
| *GRM6* | 5 | 2 |
| *PLEC* | 5 | 2 |
| *CRIPAK* | 6 | 3 |
